# Strategies for sustainable crop production in the Yangtze River Basin of China: insights from field experiments and farm surveys

**DOI:** 10.3389/fpls.2026.1933498

**Published:** 2026-10-02

**Authors:** Yalan Ji, Shuang Cheng, Zhong Li, Youzun Xu, XueYuan Sun, Huawen Zou, Yongjin Zhou

**Affiliations:** 1 Rice Research Institute, Anhui Academy of Agricultural Sciences, Hefei, Anhui, China; 2 College of Agriculture, Yangtze University, Jingzhou, Hubei, China

**Keywords:** carbon footprint (CF), economic benefit, productivity, resource use efficiency, rice-based cropping systems

## Abstract

**Introduction:**

Rice-based cropping systems in the Yangtze River Basin of China are diverse.

**Objectives:**

However, most previous studies have focused primarily on improving the yield, resource use efficiency, or reducing the carbon emissions of individual systems, with few comprehensive comparisons across different systems.

**Methods:**

To address this knowledge gap, a two-year field experiment combined with a survey of 190 farmers was conducted to evaluate the sustainability performance index (SPI) of four systems in this region: rice−fallow (RF), rice−green manure (RG), rice−wheat (RW), and rice−rapeseed (RR). Results showed that the RR and RW systems achieved higher yields than the RG and RF systems in both experiments and surveys.

**Results and conclusions:**

Average rice equivalent yields of the RR, RW, and RG systems exceeded those of the RF by 124.5%, 86.2% and 13.3%, respectively, in the experiments (RF, 9.34 t ha^-1^), and by 66.9%, 58.2%, and 2.8% in the surveys (RF, 8.96 t ha^-1^). The RR and RW systems also exhibited higher net energy, radiation use efficiency, and accumulated temperature production efficiency. Although the RR and RW systems incurred higher carbon and agricultural cost, their superior grain yield resulted in a greater net economic income, net ecosystem economic budge, and benefit-cost ratio than the RF and RG systems. Furthermore, the carbon emission intensity of the RR, RW, and RG systems in the experiments was 31.3%, 28.5%, and 8.1% lower than that of the RF (102.2 kg CO_2_eq GJ^-1^), respectively, with corresponding reductions of 22.3%, 39.4%, and 25.8% in the surveys (RF, 85.7 kg CO_2_eq GJ ^-1^). Overall, the RR and RW systems exhibited higher SPI with enhanced productivity, resource use efficiency, and economic benefits, despite comparable or marginally increased environmental impacts.

## 1 Introduction

The problems of climate change and resource scarcity are becoming more acute, which have dramatic impacts on crop production (IPCC, 2023). Porter et al. (2014) estimated global crop yield reduction due to climate change to be about 1% per decade. As the world’s population increases dramatically, the demand for food production is being put to a great test. By 2035, global crop yield will need to be increased by 116 million tons to meet the food demands of the growing population (Song et al., 2021). Increasing agricultural productivity is a crucial measure for ensuring food security; however, this process often leads to increased resource consumption and substantial carbon emission (GHG) (Wang et al., 2024; Zhang et al., 2023). Agricultural systems are important components of terrestrial ecosystems, and the agricultural production is one of the major sources of atmospheric GHG, accounting for nearly 13.8% of global emissions (IPCC, 2014). Therefore, it is crucial to improve resource use efficiency and environmental sustainability while ensuring food security (Hu et al., 2025).

The Yangtze River Basin serve as a vital production base for both grain and oil crops in China, playing a critical role in safeguarding national food security. Rice is the dominant grain crop in this region, with the rice−wheat (RW) and rice−rapeseed (RR) systems being the most extensively adopted systems. Crop rotation and intercropping are important cropping systems that enhance crop yield per unit area, improve resource use efficiency, and reduce GHG (Dong et al., 2015; Mudare et al., 2025). In recent years, with the No. 1 Central Document advocating for large-scale yield enhancement initiatives for grain and oil crops, the planting area of the RW and RR systems have expanded rapidly, which contributes to the enhancement of China’s food security. However, due to suboptimal cultivation techniques leading to low winter crop yields, a proportion of farmers in the region generally perceive winter crop cultivation as unprofitable. The large and inefficient use of agricultural inputs associated with the pursuit of high yields, such as over-application of fertilizers, has led to reduced energy efficiency, wasted agricultural resources and damaged environments (West et al., 2014; Zhang et al., 2015). Previous studies showed that the energy input of the RR system is 89.7% higher than that of rice−follow (RF) system (Huang et al., 2022). Lin et al. (2021) found that the direct GHG of the RW system are 66.7% higher than that of the RF system. Meanwhile, factors such as an aging population, labor shortages, rising farming costs, and worsening weather conditions have contributed to a growing trend of winter fallow farmland, resulting in severe underutilization of both land and light-thermal resources (Yuan and Peng, 2017). With the advocacy from governments and scholars, a growing number of farmers are willing to adopt winter green manure cultivation to enhance soil fertility and mitigate continuous cropping obstacles (Cao et al., 2024; Xiong et al., 2025). Zhong et al. (2021) found that the accumulation of methane (CH_4_) during the rice growing season in rice−green manure (RG) system was 32.95%−63.22% lower than the RF system, which was mainly due to the increase in soil permeability by drought and water rotations and the reduction of soil CH_4_ emissions.

Currently, most research focus on optimizing individual systems through improved varieties, management practices, and addition of biomaterials to obtain high yields and low GHG (Ahmad et al., 2024; Bashir et al., 2025; Bhunia et al., 2021; Kaur et al., 2025; Ma et al., 2024; Xiong et al., 2025). However, a few studies have been conducted to compare differences in various rice-based cropping systems. Winter crop adoption will necessarily increase yields compared to the RF system, but it is not clear whether increased agricultural inputs are associated with higher economic benefits, resource use efficiency, and environmental sustainability. To address these issues, we conducted a two-year (2022–2024) field experiment and a farmer survey to comprehensively assess the performance of four major rice-based cropping (RF, RG, RW, and RR) systems in the Yangtze River Basin. The objectives of this study were: 1) to determine whether the RR and RW systems could achieve higher productivity while improving resource use efficiency and economic benefits, and reducing environmental impacts compared to the RF and RG systems, and 2) to evaluate the sustainability performance index (SPI) of the four systems and identify the key factors contributing to their differences.

## 2 Materials and methods

### 2.1 Field experiment

The field experiment was carried out from 2022 to 2024 (hereafter referred to as 2022–2023 and 2023–2024) on a farmer’s field in Chaohu County, Hefei city, Anhui Province, China (31.66° N, 117.78° E) (**Figure 1**). The precipitation, daily solar radiation and temperature throughout the crop-growing season in 2022–2024 are presented in **Supplementary Figure S1**. Topsoil samples (0–20 cm) were collected for soil property analysis prior to basal fertilizer application for winter crops in 2022. The soil of the experimental site is classified as a clay loam texture. The chemical properties of the soils were as follows: organic matter of 41.55 g kg^-1^, total nitrogen of 2.10 g kg^-1^, Olsen phosphorus of 22.75 mg kg^-1^, available potassium of 275.43 mg kg^-1^, and pH of 6.44.

**Figure 1:**
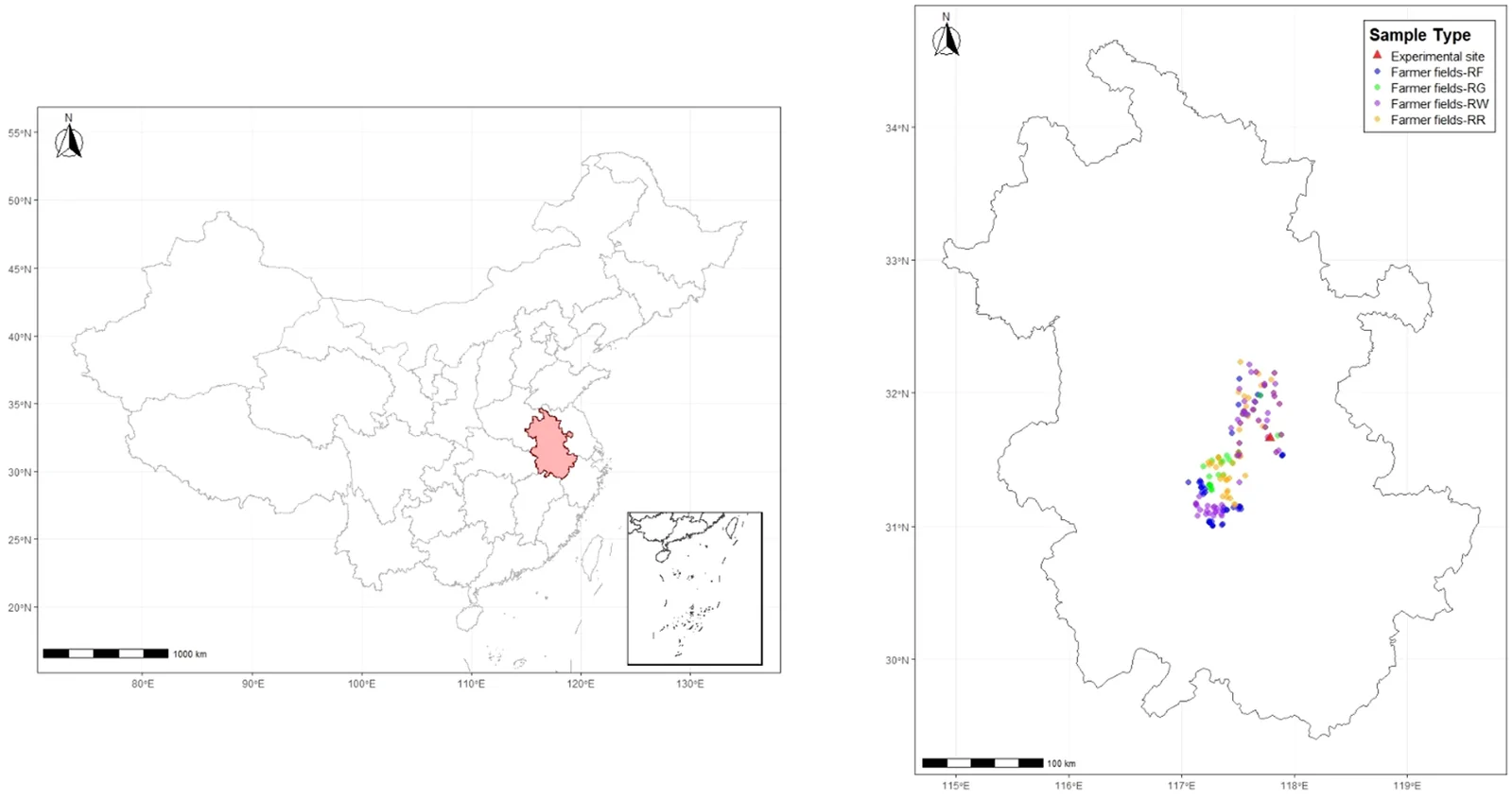
Location distribution map of experimental site and farmer fields. RF, rice−fallow; RG, rice−green manure; RW, rice−wheat; RR, rice−rapeseed.

A randomized complete block experimental design with four replications was used for the field experiment. Each plot size was 1466.7 m^2^ (48.89 m×30 m). The four major rice-based cropping systems were the RF, RG, RW, and RR systems (**Supplementary Figure S2**). Local predominant crop cultivars were selected for the experiment, N Lianyou 1998 for rice, Chinese milk vetch for green manure, Zhenmai 12 for wheat, and Fangyou 777 for oilseed rapeseed. Meanwhile, drainage and irrigation ditches were installed in the field. The same plots were maintained under the same cropping systems across both years.

In both years, pre-germinated rice seeds were sown on May 20 and mechanically transplanted on June 20 at a spacing of 16.0 cm×30.0 cm with 3−4 seedlings per hill. After sowing, the seedling bed was covered with transparent plastic film to protect the seedlings from low-temperature chilling injury. The timing of film removal was determined based on seedling growth status and ambient temperature to ensure that seedling quality at the time of transplanting was comparable between the two transplanting-date treatments. Nitrogen (N), phosphorus (P, refers to P_2_O_5_), and potassium (K, refers to K_2_O) fertilizers were applied at rates of 245 kg ha^-1^, 175 kg ha^-1^, and 220 kg ha^-1^, respectively. In wheat season, the seeds were mechanically sown on November 10 at a rate of 300.0 kg ha^-1^. N, P and K fertilizers were applied at rates of 210 kg ha^-1^, 175 kg ha^-1^, and 175 kg ha^-^¹, respectively. In rapeseed season, the seeds were mechanically sown on October 14 at a rate of 7.5 kg ha^-1^. N, P and K fertilizers were applied at rates of 270 kg ha^-1^, 200 kg ha^-1^, and 230 kg ha^-1^, respectively. Green manure was sown by hand on October 14 at a rate of 60 kg ha^-1^. Treatments with management techniques outlined in **Table 1**. Fallow fields are maintained without crops or treatments throughout the winter. After crop harvest, the original furrow pattern was disrupted during land preparation, and the straw was mechanically incorporated into the field. The environmental indicators of straw at maturity stage see **Supplementary Table S1**. Water, pest and weed management strategies were implemented in accordance with the prescribed technical guidelines for the cultivation of high-yield. The same plots were maintained under the same cropping systems across both years.

**Table 1:** The management practices for different treatment scenarios.

Scenarios	Rice season	Green manure season	Wheat season	Rapeseed season
Crop				
Name	N Lianyou 1998	Chinese milk vetch	Zhenmai 12	Fangyou 777
Sowing date (day-month)	20-May	14-Oct	10-Nov	14-Oct
Transplanting date (day-month)	20-Jun	/	/	/
Method	Mechanical transplanting	Manual broadcast sowing	Mechanical drill seeding	Mechanical drill seeding
Fertilizer				
Type	15-15-15 compound fertilizer, Urea, KCl fertilizer			
Basal and topdressing proportion	N 12:5; P 1:0; K 4:1	N 12:5; P 1:0; K 4:1	N 24:5; P 1:0; K 1:0	N 13:5; P 1:0; K 13:2
Straw				
Biomass amount (t hm^-2^)	30−47	15−18	10−15	8−10
Time (day-month)	2-Oct	14-Jun	14-Jun	30-May
Particle size (cm)	5−10	0−5	5−8	10−15

### 2.2 Farmer survey

To mitigate the limitations inherent in a single-site field experiment, a complementary farm household survey was conducted during 2022–2023. The survey employed a random sampling method to collect data on four rice-based cropping systems through questionnaire surveys of farmers from the county where the experimental fields were located and two neighboring counties (Shen et al., 2020). A total of 33 townships and 103 villages across the three counties were involved in the survey. In each of the four systems, 25 farming were sampled, yielding a total of 300 farmers across all systems. The numbers of households that refused to participate in the survey were 15, 20, 12, and 10 for the RF, RG, RW, and RR systems, respectively. Cases of complete crop failure, drought damage, and missing record information were also observed. The data included field locations, area, crop yield, cultivation management practices, and various agricultural inputs (**Supplementary Table S2**). After excluding the uncertain responses (harvest failure, drought, lack of recorded information, and et. al), the final sample of this study comprised 190 famers (50 RF system famers, 30 RG system famers, 55 RW system famers, and 55 RR system famers) (**Figure 1**).

### 2.3 Measurements and calculations

#### 2.3.1 Yield and yield attributes

In each plot of field experiment, a uniform growing area of four 5 m^2^ were selected at maturity, with border effects deliberately avoided, for harvesting and threshing the grains of rice, wheat, or rapeseed, followed by sun-drying and adjustment to standard moisture content for actual grain yield calculations. Simultaneously, plants from a uniformly growing area of 0.5 m^2^ were collected from each plot. For rice and wheat, the plants were separated into stems, leaves, and panicles, while for rapeseed, they were divided into stems, pods (seedless), and seeds. The samples were then packaged and oven-dried at 105 °C for 30 minutes for de-enzyming, followed by drying at 80 °C to constant weight. Subsequently, they were weighed to calculate the total dry matter accumulation and harvest index at maturity. Carbon (C) and nitrogen (N) concentrations in various tissues and organs at maturity were determined using an isotope ratio mass spectrometer (Elementar Trading Co., Ltd, Germany), and the carbon and nitrogen contents were calculated based on the corresponding dry matter weights. Additionally, during the mature stage, employ the five-point sampling method to collect soil samples from 10 cm to 15 cm below the plant roots, placing them into sterile bags. The freshly collected soil samples are tested for pH using the potentiometric method (Agricultural Industry Standard of the People’s Republic of China, NY/T 1377−2007). For the farmer survey, crop yield was calculated by converting the farmer-reported grain yield and moisture content to a standard moisture level.

Rice equivalent yield (t ha^-1^), and energy yield (GJ ha^-1^) were calculated as the sum of all harvested crops within the annual system. The specific calculation formulas are as follows (Equations 1, 2):

(1) $$\text{Rice equivalent yield}=Y_{i}\times P_{i}/P_{rice}$$

(2) $$\text{Energy yield}=Y_{i}\times E_{i}$$

Where *Y_i_* (t ha^-1^) and *P_i_* (US$ kg^-1^) represent grain yield adjusted to standard moisture content (rice 13.5%, wheat 13.0%, rapeseed 8%) and selling price for rice, wheat or rapeseed, respectively. *P_rice_* (US$ kg^-1^) is the selling price of rice grain. *E_i_* (MJ kg^-1^) represents the energy equivalent coefficient of rice, wheat, and rapeseed (**Table 2**).

**Table 2:** The energy equivalent coefficients and prices of various agricultural inputs and outputs.

Items	Unit	Energy equivalent (MJ unit^-1^)	Reference	Price (US$ unit^-1^)
Input				
Machinery	h	62.7	Nabavi et al., 2014	1.43
Diesel	kg	56.31	Nabavi et al., 2014	0.99
Nitrogen (N)	kg	66.1	Nabavi et al., 2014	0.67
Phosphorus (P_2_O_5_)	kg	12.4	Nabavi et al., 2014	0.18
Potassium (K_2_O)	kg	11.1	Nabavi et al., 2014	0.82
Electricity	KWh	11.93	Kuswardhani et al., 2013	0.15
Insecticide	kg	160	Truong et al., 2017	18.67
Fungicide	kg	216	Singh et al., 2019	114.87
Herbicide	kg	238	Pathak and Bining, 1985	77.54
Rice seed	kg	14.7	Pathak and Bining, 1985	8.61
Wheat seed	kg	15.7	Pathak and Bining, 1985	0.66
Rapeseed	kg	29.2	Unakitan et al., 2010	17.23
Green manure seed	kg	/	/	4.51
Labor	h	1.96	Pathak and Bining, 1985	2.9
Output				
Rice grain	kg	14.7	Eskandari and Attar, 2015	0.37
Wheat grain	kg	14.7	Singh et al., 2022	0.34
Rapeseed grain	kg	28.3	Unakitan et al., 2010	0.91

Prices for all agricultural inputs, except seeds, were obtained from the *China Yearbook of Agricultural Price Survey*. The prices were derived as follows: rice seeds, wheat seeds, rapeseed, and green manure seeds from local market prices; rice grains and wheat grains from the two-year average of the National Development and Reform Commission’s minimum purchase prices; and rapeseed grain from its two-year average market price at harvest. The CNY/USD exchange rate averaged 6.9648 during the 2022-2024 study period, sourced from the Chinese State Administration of Foreign Exchange (http://www.safe.gov.cn/).

#### 2.3.2 Resource utilization

Agricultural inputs of four rice-based cropping systems were collected, including machinery, diesel, chemical fertilizers, electricity, insecticide, fungicide, herbicide, seed, and labor. The amounts of various agricultural inputs used in the experiment and survey are presented in **Table 3**.

**Table 3:** The agricultural inputs of four rice-based cropping systems.

Items	Unit ha^-1^	Experiment 2022–2023				Experiment 2023–2024				Survey			
		RF	RG	RW	RR	RF	RG	RW	RR	RF	RG	RW	RR
Machinery	h	13.6	17.0	20.5	27.3	13.6	17.0	20.5	27.3	28.1	35.3	66.9	60.6
Diesel	kg	120.0	150.0	180.0	240.0	120.0	150.0	180.0	240.0	144.0	180.7	342.5	310.2
Nitrogen (N)	kg	245.0	245.0	455.0	515.0	245.0	245.0	455.0	515.0	234.0	194.3	379.6	411.6
Phosphorus (P_2_O_5_)	kg	175.0	175.0	350.0	375.0	175.0	175.0	350.0	375.0	76.2	57.2	190.0	193.0
Potassium (K_2_O)	kg	220.0	220.0	395.0	450.0	220.0	220.0	395.0	450.0	120.3	112.2	247.0	280.2
Electricity	KWh	526.3	526.3	789.5	789.5	526.3	526.3	789.5	789.5	527.0	538.5	763.9	596.4
Insecticide	kg	3.3	3.3	4.8	5.3	3.9	3.2	5.4	5.5	3.9	3.2	2.4	2.3
Fungicide	kg	0.7	0.7	1.5	1.6	0.9	0.8	2.1	2.5	3.7	3.3	2.2	1.5
Herbicide	kg	2.6	2.8	3.5	4.3	2.8	3.0	5.3	6.2	2.7	3.2	3.6	3.0
Rice seed	kg	28.5	28.5	28.5	28.5	28.5	28.5	28.5	28.5	26.6	24.3	30.5	31.8
Wheat seed	kg	0.0	0.0	300.0	0.0	0.0	0.0	300.0	0.0	0.0	0.0	282.2	0.0
Rapeseed	kg	0.0	0.0	0.0	7.5	0.0	0.0	0.0	7.5	0.0	0.0	0.0	7.9
Labor	h	300.0	375.0	450.0	450.0	300.0	375.0	450.0	450.0	27.7	28.8	37.4	40.3

RF, rice−fallow; RG, rice−green manure; RW, rice−wheat; RR, rice−rapeseed.

Energy output (MJ ha^-1^) is equivalent to the energy yield (Equation 3).

(3) $$\text{Energy input }\left({\text{MJ ha}}^{-1}\right)=\sum \left(Q_{i}\times \epsilon_{i}\right)$$

Where *Q_i_* is the amount of each agricultural input (kg ha^-1^) in the four rice-based cropping systems (no agricultural inputs in the fallow season); and *ϵ_i_* refers to the energy equivalent coefficients of each agricultural input (MJ unit^-1^, **Table 2**).

Net energy (GJ ha^-1^) and energy productivity (MJ MJ^-1^) were calculated as follows (Kaab et al., 2019) (Equations 4, 5):

(4) Net energy=Energy output−Energy input

(5) Energy productivity=Energy yield/Energy input

Radiation production efficiency (RPE, MJ MJ^-1^) and accumulated temperature production efficiency (TPE, MJ ha^-1^ °C^-1^ d ^-1^) of each system were calculated as follows (Equations 6, 7):

(6) RPE=Energy yield/Accumulated radiation

(7) TPE=Energy yield/Effective cumulative temperature

Where the accumulated radiation (MJ m^-2^) and effective cumulative temperature (°C d^-1^) are the sum of the daily incident solar radiation and effective temperature (rice>10 °C, wheat and rapeseed>0 °C), respectively, during the crop growth season (**Supplementary Figure S1**).

#### 2.3.3 Environmental impact

The LCA (life cycle assessment) method was used to analyze the carbon footprint (CF) of different rice-based cropping systems and to quantify the GHG during the whole life cycle process of each growing season within the system. The annual system boundary includes the whole life cycle process from winter crops sowing to rice harvesting. The LCA boundary should be explicitly defined, including fertilizer and pesticide production, machinery manufacture, seed production, irrigation, electricity generation, transport, and residue management were included (Equations 8–10).

(8) $$\text{CF}={\text{GHG}}_{\text{indirect}}+{\text{GHG}}_{\text{direct}}$$

(9) $${\text{GHG}}_{\text{indirect}}=\sum \left(Q_{i}\times \mu_{i}\right)$$

(10) $${\text{GHG}}_{\text{direct}}={\text{GHG}}_{{\text{CH}}_{4}}\times 28+{\text{GHG}}_{{\text{N}}_{2}\text{o}}\times 273$$

Where CF is carbon footprint (kg CO_2_eq ha^-1^); GHG_indirect_ (kg CO_2_eq ha^-1^) and GHG_direct_ (kg CO_2_eq ha^-1^) are the indirect and direct GHGs, respectively; *Q_i_* and *µ_i_* represent the amount of agricultural input and its corresponding carbon dioxide (CO_2_) equivalent coefficient, respectively (**Supplementary Table S3**); ${\text{GHG}}_{{\text{CH}}_{4}}$ (kg ha^-1^) and ${\text{GHG}}_{{\text{N}}_{2}\text{o}}$ (kg ha^-1^) are cumulative GHG of CH_4_ and nitrous oxide (N_2_O), respectively. Where 273 and 28 are the global warming potential coefficients for N_2_O and CH_4_ from a period of 100 years, respectively (IPCC, 2021).

The field experiment employed the static chambers method to collect direct CH_4_ and N_2_O emissions throughout the crop growing seasons (Wang et al., 2024). The gas was collected at weekly intervals throughout the rice season (sampling time 9:00−11:00) and at 10−14 days intervals during the winter season. The static chamber consists of a case and a base made of stainless steel. The case has dimensions of 60 cm×40 cm×120 cm. The case has a heat-insulating layer, a fan, an air extraction port, and a digital thermometer. The base measures 60 cm×40 cm×8 cm with a 2.5 cm internal diameter of the groove. One month before the experiment, the bases were buried in the middle of the plots (four in each plot), placed horizontally to a depth of 25 cm−30 cm in the soil. And the upper end was used as a sealing gutter to confine the gas discharge area and to seal and fix the gas harvesting tanks, which were kept immobile at a later stage. To prevent the soil from being disturbed during gas collection, build a bridge for gas collection. Gas extraction and other agricultural operations do not trample the soil within 15 m of the static box. First, when the air in the stationary box was well mixed and left to stand for 6 min, the first gas collection was started with a 50 mL syringe. After which the gas was collected every 6 min, for a total of 5 times and the temperature inside was recorded at each collection. The collected samples were analyzed by gas chromatograph (Agilent 7890 A model, Agilent Technologies, Inc., USA). Cumulative emissions of gases during the growing seasons are shown below. The cumulative emissions (kg ha^-1^) of gases during the growing seasons are shown below (Equation 11).

(11) $$\text{Cumulative emmisions}=\sum \left[\left(F_{i}+F_{i+1}\right)/2\times 10^{-3}\times d\times 24\times 10\right]$$

Where *F_i_* and *F_i+1_* are the fluxes of gases emitted during two consecutively adjacent sampling periods (mg m^-2^·h^-1^); *d* is the number of days between two consecutively adjacent sampling periods.

To mitigate measurement errors arising from the large number of farm households, the direct GHG emissions in the household survey were derived from DNDC (De Nitrification-De Composition) model data. The DNDC model is a biogeochemical model that describes soil carbon and nitrogen cycles and carbon emission fluxes in agricultural systems (http://www.dndc.sr.unh.edu/; Gilhespy et al., 2014). Daily meteorological data for each weather station required by the DNDC model were obtained from the meteorological bureau, processed into the format required by the model, and used to construct a meteorological database, with stations assigned to the nearest locations geographically. Model parameter calibration is shown in **Supplementary Table S4**. As a predictive tool, the model predictions must be validated using field measurements. In this study, data from the four cropping pattern treatments in the first year of field experiments were selected to calibrate the DNDC model, while data from the second year were used for model validation. Model accuracy was evaluated by calculating the model efficiency (ME), and the correlation between simulated and observed values was tested using the coefficient of determination (r²) (Equations 12, 13) (Yang et al., 2014).

(12) $$\text{ME}=1-\frac{\sum_{i=1}^{n}{\left(P_{i}-M_{i}\right)}^{2}}{\sum_{i=1}^{n}{\left(M_{i}-\bar{M}\right)}^{2}}$$

(13) $${\text{r}}^{2}={\left(\frac{\sum_{i=1}^{n}\left(M_{i}-\bar{M}\right)\left(P_{i}-\bar{P}\right)}{\sqrt{\sum_{i=1}^{n}{\left(M_{i}-\bar{M}\right)}^{2}}\sqrt{\sum_{i=1}^{n}{\left(P_{i}-\bar{P}\right)}^{2}}}\right)}^{2}$$

where *P_i_* is the simulated value; *M_i_* is the observed value; i is the number of samples; $\bar{M}$ is the mean of the observed values; and $\bar{P}$ and *M* are the means of the simulated and observed values, respectively. The ME value can be either positive or negative. Specifically, a positive value indicates that the simulated values describe the trends in the measured data better than the mean of the measured values, and a value closer to 1 signifies greater modeling efficiency. A negative value indicates that the simulated values describe the data less effectively than the mean of the measured values.

The carbon emission intensity (GHGI, kg CO_2_eq GJ^-1^) was calculated as follows (Equation 14):

(14) GHGI=CF/Energy yield

#### 2.3.4 Economic profitability

Net ecosystem economic budget (NEEB, US$ ha^-1^), net economic income (NEI, US$ ha^-1^), and the benefit-cost ratio ecoefficiency were calculated as follows (Equations 15–20):

(15) NEEB=NEI−Cost carbon

(16) NEI=Income grain yield−Agriculture cost

(17) Benefit-cost ratio=Income grain yield/Agriculture cost

(18) $$\text{Income grain yield}\left(\text{US}\${\text{ ha}}^{-1}\right)=\text{Grain yield}\times \text{Grain price}$$

(19) $$\text{Agriculture cost}\left(\mathrm{US}\${\text{ ha}}^{-1}\right)=\sum \left(M_{i}\times \text{Unit prices}\right)$$

(20) $$\text{Cost carbon}\left(\mathrm{US}\${\text{ ha}}^{-1}\right)=\text{CF}\times \text{Carbon price}$$

Where the *M_i_* and unit prices are the input materials and their corresponding unit prices (**Table 2**). Carbon price means the price of GHG, which is specifically 11.09 US$ CO_2_-eq t^-1^ in China (Deng et al., 2024). All price data were sourced from reputable authorities, namely the National Bureau of Statistics of China (https://www.stats.gov.cn/) and the National Food and Strategic Reserves Administration of China (http://www.lswz.gov.cn), thereby ensuring data reliability.

#### 2.3.5 Sustainable evaluation

The concept of sustainability lacks a clear definition, as its evaluation methods vary inconsistently depending on the standpoint of the evaluator, and the concept itself is inherently contentious. To determine which paddy cropping system can more effectively address the agricultural production challenges, this study assumes that the implications of food security, resource utilization, environmental impact, and economic profitability are aligned. This allows for further exploration of the advantages of different cropping systems in specific dimensions, as well as strategies to mitigate their shortcomings in other dimensions, thereby achieving greater comprehensive benefits in rice-based cropping systems.

The study assesses four key aspects of sustainability of different rice-based cropping systems by normalizing and weighting similar indicators to draw more comprehensive and convincing conclusions (Cui et al., 2021; Xu et al., 2022; Yang et al., 2023). The main components are: 1) Food security (rice equivalent yield and energy yield); 2) Resource utilization (net energy, energy productivity, RPE, and TPE); 3) Environmental impact (CF and GHGI); 4) Economic profitability (NEEB, NEI, and benefit-cost ratio). The specific algorithm was as follows (Equations 21–24):

(21) $$\Phi^{\prime }=\left(\Phi_{max}-\Phi \right)/\left(\Phi_{max}-\Phi_{min}\right)\times 100$$

(22) $$\Phi I=\text{INT}\left(1-\Phi^{\prime }\right)/20\%+1,\text{ if }\Phi^{\prime }=0,\text{ then }\Phi I=5$$

where *Ф’* refers to the normalized performance score of these calculated indicators. *Ф* represents the initial values of the calculated indicators corresponding to the four key aspects. *Ф_max_* and *Ф_min_* denote the best and worst performance, respectively. For instance, for energy yield, *Ф_max_* and *Ф_min_* represent the maximum and minimum energy yield values. In contrast, for CF, *Ф_max_* and *Ф_min_* indicate the minimum and maximum values. *ФI* is the score of each indicator.

(23) $$\lambda_{i}=\sum \left(\gamma_{i}\times \Phi I_{i}\right)$$

(24) $$\text{SPI}=\sum \left(\theta_{i}\times \lambda_{i}\right)$$

where *λ_i_* and *ФI_i_* are the score of each aspect and indicator. *γ_i_* and *θ_i_* are the weight factor of each indicator and target. The value of *γ_i_* in this study is 1/n, where n is the number of selected indicators under that aspect. Specifically, these weights are 1/2 for food security, 1/4 for resource utilization, 1/2 for environmental impact, and 1/3 for economic profitability. The value of *θ_i_* set to 1/4 in this study. SPI is the sustainability performance index of each system. A higher SPI indicates greater sustainability of the systems. The specific steps of the algorithm are described in Cui et al. (2021) and Xu et al. (2024a).

### 2.4 Statistical analysis

Data preprocessing was performed using Microsoft Excel 2021. All statistical inferences were conducted utilizing Statistix 9 (Analytical Software, Tallahassee, FL, USA). The experiment was conducted using a completely randomized block design with four plots and four replications per plot. Data were analyzed using a linear mixed model (LMM) to account for field-scale spatial heterogeneity and the non-independence of observations within blocks. In each year, data were analyzed using a Randomized Complete Block AOV model, with significance set at p<0.05. When the overall treatment effect was significant, data were analyzed using a Split-Plot Design AOV model, with cropping system as the main-plot factor and year as the sub-plot factor. Mean comparisons were performed at the p<0.05 and p<0.01significance levels. Prior to model fitting, the normality of residuals was rigorously assessed using the Kolmogorov-Smirnov test and the Shapiro–Wilk test on standardized residuals. Data were considered to meet model assumptions when p>0.05 for both tests. The comparison of treatment means being based on the least significant difference (LSD) test at the probability levels of 0.05. The graphical representations were generated using the software package Origin Pro 2025 and R 4.4.3.

## 3 Results

### 3.1 Food security

There were significant differences in rice equivalent yield among the cropping systems in both the experiments and surveys (**Figure 2**). Averaged across two cycles of experiments, the RR system demonstrated the highest rice equivalent yield of 20.97 t ha^-1^ among all systems, which was significantly increased by 124.5%, 98.1%, and 20.6% compared to that of the RF, RG, and RW systems, respectively. Survey data on rice yield equivalents across the four cropping systems aligned with the experimental findings, demonstrating the same pattern: RR>RW>RG>RF. For energy yield, both the RR and RW systems outperformed the other systems in both experiments and surveys, although no significant difference was observed between them. Averaged across two cycles of experiments, the energy yield of the RF system was significantly lower than that of the RG, RW, and RR systems by 13.3%, 93.3%, and 99.3%, respectively; these reductions magnitudes were 2.8%, 63.1%, and 52.9% in the survey. Winter cultivation of wheat, rapeseed, or green manure significantly increased the grain yield, total dry weight, and total nitrogen uptake of the subsequent rice crop (**Figure 2**; **Supplementary Table S1**). Compared with the RF system, the RG, RW, and RR systems increased rice grain yield by 13.3%, 5.1%, and 8.5%, respectively; total dry weight by 33.1%, 15.2%, and 28.2%, respectively; and total nitrogen uptake by 38.4%, 16.0%, and 42.3%, respectively.

**Figure 2:**
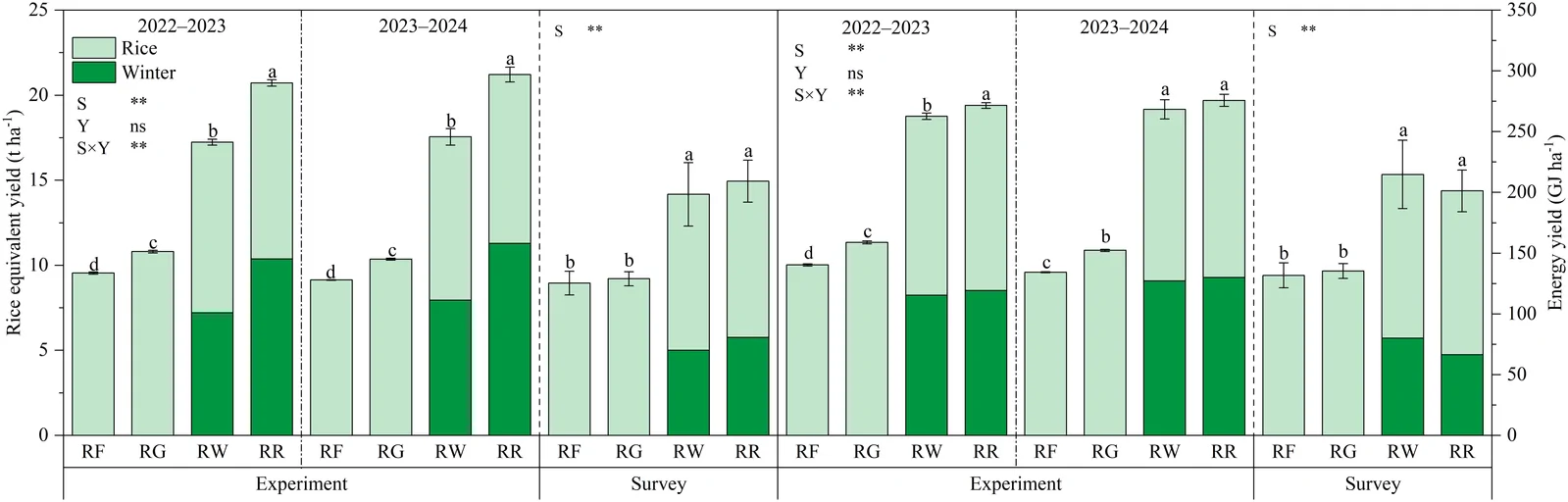
The rice equivalent yield and energy yield of four rice-based cropping systems. S, system; Y, year; RF, rice−fallow; RG, rice−green manure; RW, rice−wheat; RR, rice−rapeseed. Within a group for each experimental year or survey, different letters are significantly different according to LSD (0.05). ns, not significant at 0.05 probability level; **significant at the 0.01 probability level, respectively.

### 3.2 Resource utilization

Both experimental and survey data indicated that the energy inputs for the RW and RR systems were significantly higher than those for the RF and RG systems. Averaged across two cycles of experiments, the RF system had the lowest energy inputs, which was 5.2%, 45.7%, and 48.8% lower than that of the RG, RW and RR systems, respectively (**Table 4**). Among all the inputs in experiments and surveys, nitrogen fertilizer had the greatest contribution (35.7% to 47.0%) to energy inputs, followed by diesel fuel (18.2% to 28.3%), and electricity (11.3% to 17.9%) (**Figure 3**). Compared to the RF system, the RR, RW, and RG systems, with their higher energy output, exhibited net energy increases of 100.8%, 97.2%, and 16.2% in the experiment, and 45.4%, 53.1%, and 4.6% in the survey, respectively. By contrast, the energy productivity variations among four systems in the experiments and surveys were inconsistent. This is primarily because the differences in rice yield equivalent among the systems were smaller in the surveys than in the experiments. In the experiments, the RPE and TPE of the RR and RW systems were significantly higher than those of the RG and RF systems, a result that was further confirmed by the survey.

**Table 4:** The resource utilization indicators of four rice-based cropping systems.

Source	Year (Y)	System (S)	Energy input	Energy output	Net energy	Energy productivity	RPE	TPE
			(MJ ha^-1^)	(MJ ha^-1^)	(MJ ha^-1^)	(MJ MJ^-1^)	(MJ MJ^-1^)	(MJ ha^-1^ °C^-1^ d^-1^)
Experiment	2022–2023	RF	37001.0 b	140313.7 d	103312.7 d	3.79 c	0.0033 c	28.95 d
		RG	39075.8 b	158862.3 c	119786.5 c	4.07 a	0.0037 b	32.78 c
		RW	68461.5 a	262645.7 b	195085.5 b	3.89 b	0.0064 a	57.74 a
		RR	72066.6 a	271552.5 a	199486.0 a	3.77 c	0.0065 a	56.03 b
	2023–2024	RF	37191.7 b	134304.5 c	97112.8 c	3.61 c	0.0027 c	26.84 d
		RG	39146.3 b	152278.0 b	113131.7 b	3.89 ab	0.0031 b	30.43 c
		RW	68225.1 a	268298.6 a	200073.5 a	3.93 a	0.0057 a	56.93 a
		RR	72718.4 a	275733.0 a	203014.6 a	3.79 b	0.0058 a	55.10 b
	Analysis of variance							
	S		**	**	**	**	**	**
	Y		ns	ns	ns	**	**	**
	S×Y		**	**	**	**	ns	ns
Survey		RF	36702.7 c	131650.3 c	94947.6 d	3.96 a	0.0025 b	25.63 b
		RG	35994.0 c	135350.3 c	99356.3 c	3.91 a	0.0025 b	26.35 b
		RW	69457.3 a	214780.6 a	145323.3 a	3.33 b	0.0040 a	41.82 a
		RR	63258.5 b	201269.9 b	138011.4 b	3.41 b	0.0038 a	39.19 a
	Analysis of variance							
	S		**	**	**	**	**	**

RF, rice−fallow; RG, rice−green manure; RW, rice−wheat; RR, rice−rapeseed; RPE, radiation production efficiency; TPE, accumulated temperature production efficiency. Within a column for each experimental year or survey, means followed by different letters are significantly different according to LSD (0.05). ns, not significant at 0.05 probability level; * and ** are significant at the 0.05 and 0.01 probability levels, respectively.

**Figure 3:**
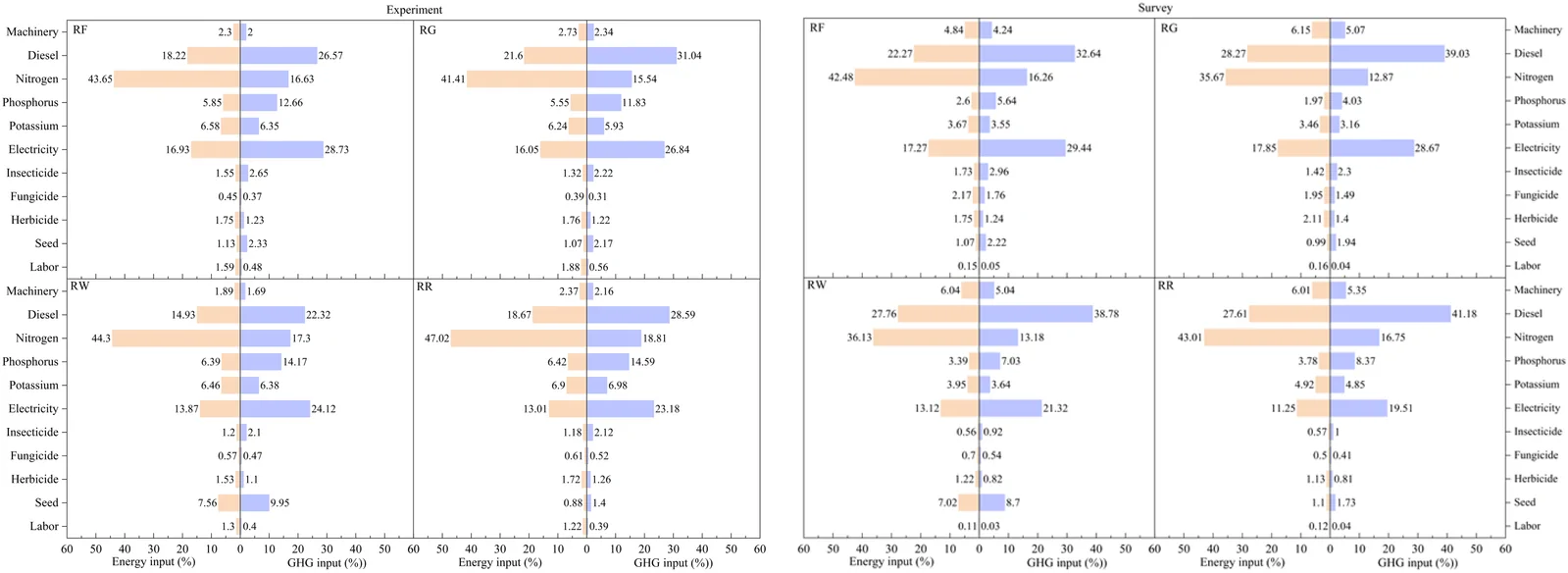
Distribution of energy input (%) and greenhouse gas (GHG) input (%) from different agricultural inputs of the four rice-based cropping systems. RF, rice−fallow; RG, rice−green manure; RW, rice−wheat; RR, rice−rapeseed.

### 3.3 Environmental impact

By simulating the monthly dynamics of cumulative CH_4_ and N_2_O emissions in 2023 under the four cropping systems using the DNDC model, we found a good fit between the observed and simulated values (**Supplementary Figure S3a**). Through calibration, the model parameters for four systems were determined, with detailed parameter settings presented in **Supplementary Table S5**. Using the calibrated model, the monthly dynamics of cumulative CH_4_ and N_2_O emissions in 2024 were simulated, and the results fell within an acceptable range, permitting further simulations (**Supplementary Figure S3b**).

Regardless of the cumulative emissions of CH_4_ or N_2_O, the changing trends of the four systems remained consistent over the two cycles (**Figure 4**). Averaged across two cycles, the RW system had the highest cumulative CH_4_ emissions, which were 23.4%, 20.5%, and 2.3% higher than those of the RF, RG, and RR systems in the experiment, and 39.2%, 29.7%, and 5.3% in the survey, respectively, respectively. However, the RW system had the lowest cumulative N_2_O emissions (0.5 g m^-2^), which were 26.3%, 15.8%, and 5.9% lower than those of the RF, RG and RR systems in the experiment, respectively. In the survey, the RR system exhibited the greatest cumulative N_2_O emissions, which was 43.2%, 99.6%, and 53.5% greater than those recorded under the RF, RG, and RW systems, respectively. The CH_4_ emission mainly occurred in rice-growing seasons, whereas N_2_O emissions were primarily observed in upland crops. Averaged across two cycles of experiments, the RF system had the lowest energy inputs (2253.4 kg CO_2_eq ha^-1^), which was 6.6%, 44.0%, and 46.2% lower than that of the RG, RW and RR systems, respectively (**Figure 3**). Among the indirect GHG emissions associated with agricultural inputs across the four systems in the experiments and surveys, diesel fuel was the largest contributor, accounting for 18.2% to 41.2%, followed by electricity (19.5% to 29.4%), nitrogen fertilizer (12.9% to 18.8%), and phosphorus fertilizer (4.0% to 14.6%).

**Figure 4:**
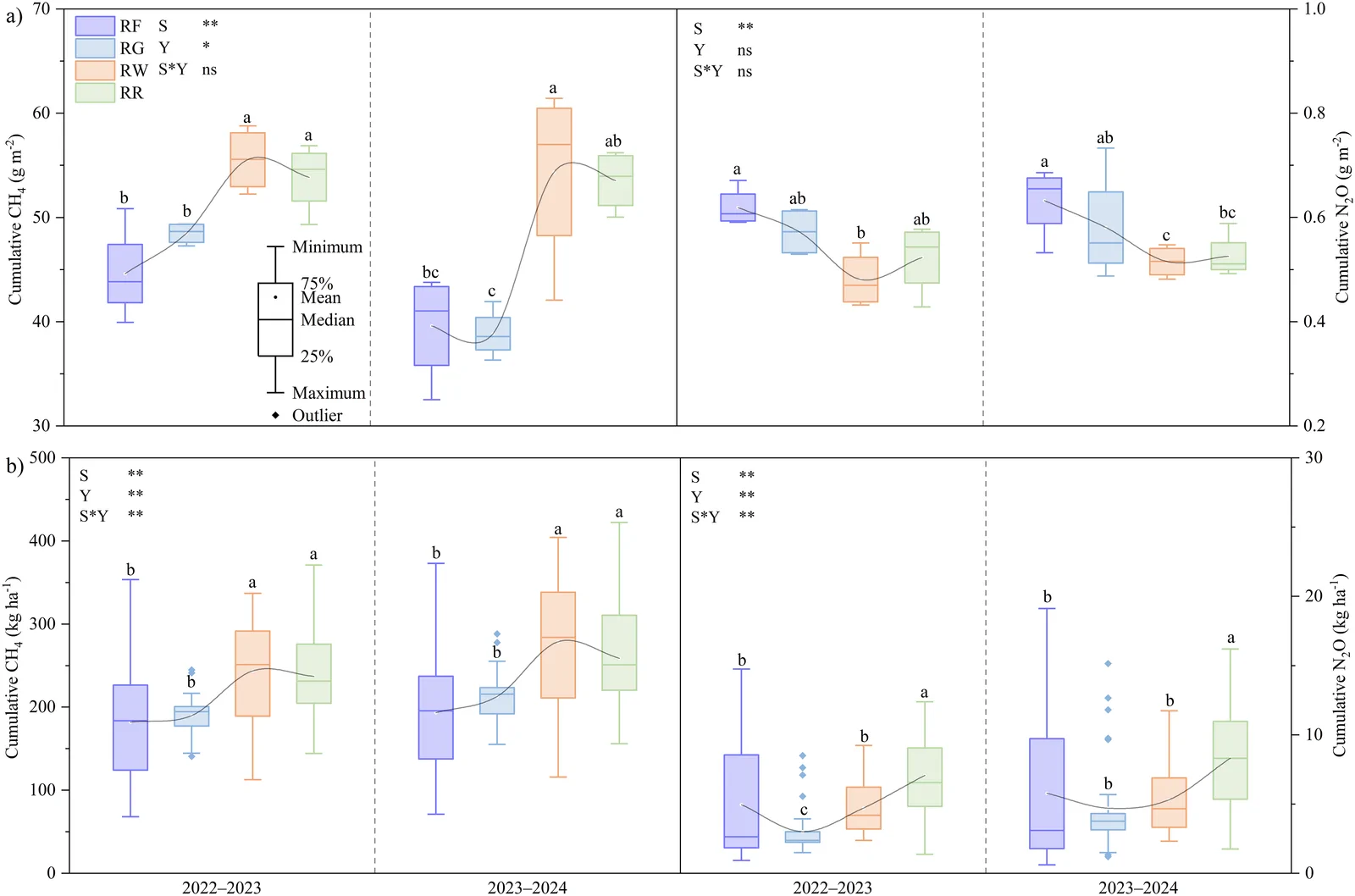
The cumulative of CH_4_ and N_2_O of the four rice-based cropping systems in experiment **(a)** and survey **(b)**. RF, rice−fallow; RG, rice−green manure; RW, rice−wheat; RR, rice−rapeseed. Within a group for each experimental year, different letters are significantly different according to LSD (0.05). ns, not significant at 0.05 probability level; * and **, significant at the 0.05 and 0.01 probability levels, respectively.

The CF of the RW and RR systems were significantly higher than that of the RF and RG systems (**Figure 5**). Averaged across two cycles of experiments, the CF of the RF system was lower than that of the RG, RR, and RW systems by 4.1%, 27.7%, and 27.0%, respectively; the RG system was lower than that of the RF, RR, and RW systems by 20.3%, 18.6%, and 29.7% in the survey, respectively. Overall, the greatest contributor to CF was CH_4_ emission. GHGI was significantly affected by system (**Figure 6**). Specifically, both experimental and survey data indicate that the RW and RR systems exhibited a significantly lower GHGI than the RF and RG systems, a trend that was inversely related to their CF.

**Figure 5:**
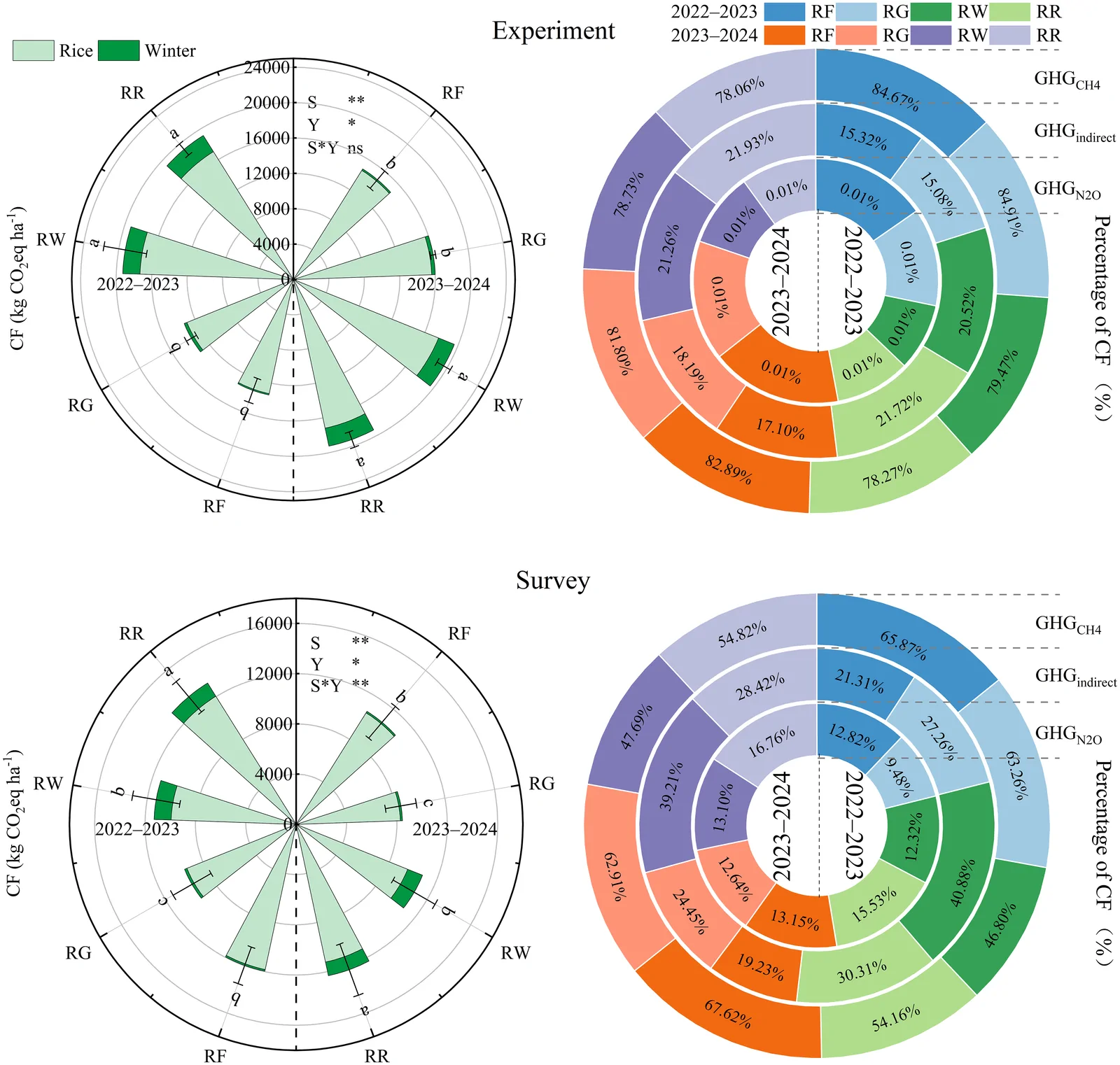
The carbon footprint (CF), and percentage of CF of the four rice-based cropping systems. RF, rice−fallow; RG, rice−green manure; RW, rice−wheat; RR, rice−rapeseed; ${\text{GHG}}_{{\text{CH}}_{4}}$, cumulative GHG emissions of CH_4_; ${\text{GHG}}_{{\text{N}}_{2}\text{O}}$, cumulative GHG emissions of N_2_O; GHG_indirect_, cumulative GHG emissions of indirect. Within a group for each experimental year or survey, different letters are significantly different according to LSD (0.05). ns, not significant at 0.05 probability level; * and **, significant at the 0.05 and 0.01 probability levels, respectively.

**Figure 6:**
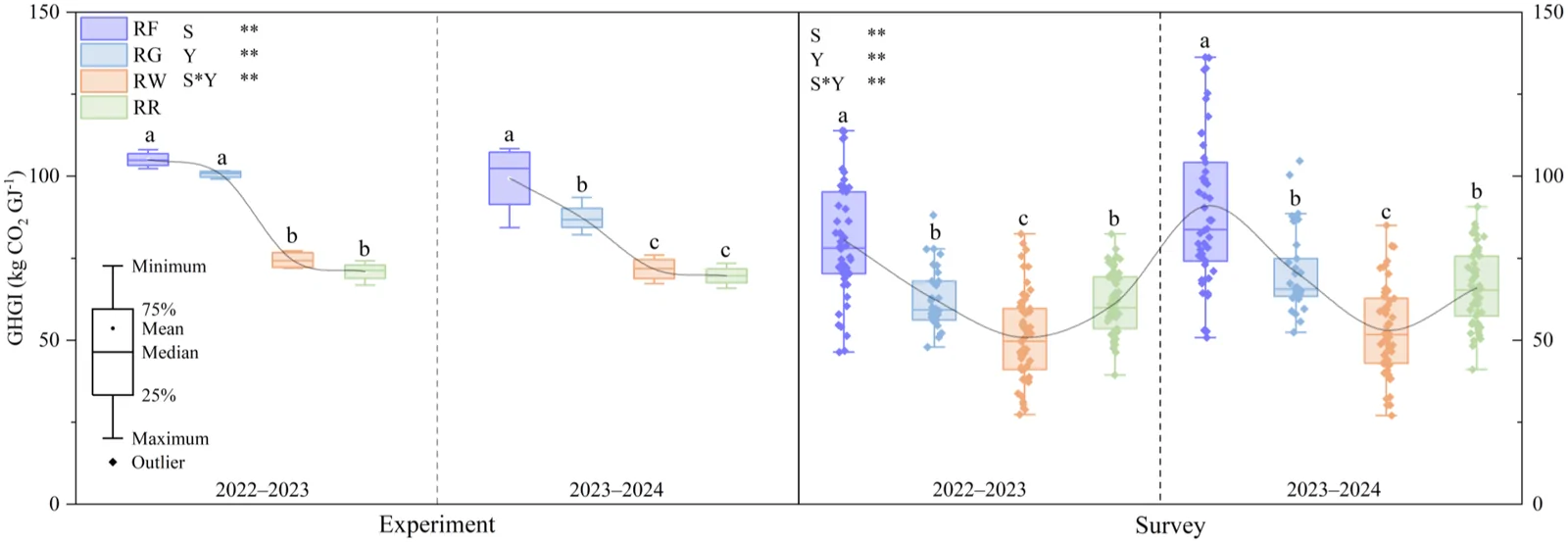
The carbon emission intensity (GHGI) of four rice-based cropping systems. S, system; Y, year; RF, rice−fallow; RG, rice−green manure; RW, rice−wheat; RR, rice−rapeseed. Within a group for each experimental year or survey, different letters are significantly different according to LSD (0.05). ns, not significant at 0.05 probability level; **, significant at the 0.01 probability level, respectively.

### 3.4 Economic profitability

The RW and RR systems exhibited higher agricultural and carbon cost than the RF and RG systems in both experiments and surveys (**Table 5**). Notably, agricultural costs consistently surpassed carbon costs across all systems. The higher income grain yield from the RR and RW systems resulted in significantly greater NEI and NEEB compared to the RF and RG systems. Averaged across two cycles of experiments, the NEEB of the RR system was 69.2%, 64.0%, and 28.9% higher than that of the RF, RG, and RW systems, respectively. Further analysis showed that the benefit-cost ratio of the RR system was 22.9%, 22.1%, and 12.4% higher than that of the RF, RG, and RW systems, respectively. Farm surveys revealed that the NEI, NEEB, and benefit-cost ratio of the RR system were 13.3%–49.9%, 14.5%–51.4%, and 9.8%–19.0% higher than those of the other systems, respectively.

**Table 5:** The economic profitability indicators of four rice-based cropping systems.

Source	Year (Y)	System (S)	Cost carbon	Cost agriculture	Income grain yield	NEI	NEEB	Benefit-cost ratio
			(US$ ha^-1^)	(US$ ha^-1^)	(US$ ha^-1^)	(US$ ha^-1^)	(US$ ha^-1^)	
Experiment	2022–2023	RF	175.4 b	2050.8 b	3531.7 d	1480.9 d	1305.5 d	1.72 c
		RG	190.2 b	2312.7 b	3998.6 c	1551.4 c	1495.6 c	1.73 c
		RW	232.8 a	3294.2 a	6375.5 b	2927.3 b	2848.6 b	1.94 b
		RR	229.1 a	3475.3 a	7668.5 a	4193.2 a	3964.1 a	2.21 a
	2023–2024	RF	158.8 b	2103.5 b	3380.5 d	1277.0 c	1118.1 d	1.61 c
		RG	158.2 b	2342.3 b	3832.8 c	1382.8 c	1332.4 c	1.64 c
		RW	229.3 a	3517.5 a	6493.6 b	2825.4 b	2746.8 b	1.85 b
		RR	228.4 a	3717.4 a	7847.7 a	4130.2 a	3901.8 a	2.11 a
	Analysis of variance							
	S		**	**	**	**	**	**
	Y		*	ns	ns	**	**	**
	S×Y		ns	**	**	ns	ns	ns
Survey		RF	152.3 b	1542.3 b	3313.6 b	1771.3 d	1616.0 c	2.42 c
		RG	161.2 b	1516.0 b	3406.8 b	1890.7 c	1729.5 c	2.43 c
		RW	219.5 a	2176.6 a	5242.7 a	3066.1 b	2846.5 b	2.69 b
		RR	208.4 a	1993.2 a	5529.3 a	3536.1 a	3327.7 a	2.99 a
	Analysis of variance							
	S		**	**	**	**	**	**

RF, rice−fallow; RG, rice−green manure; RW, rice−wheat; RR, rice−rapeseed; NEI, net economic income; NEEB, net ecosystem economic budget. Within a column for each experimental year or survey, means followed by different letters are significantly different according to LSD (0.05). ns, not significant at 0.05 probability level; * and ** are significant at the 0.05 and 0.01 probability levels, respectively.

### 3.5 Sustainable evaluation

The SPI values of the RR and RW systems were higher than those of the RF and RG systems across both experiments and surveys. Specifically, in the experimental results, the RR system had the highest SPI of 4.31, which was 65.2%, 46.4%, and 6.3% higher than the values of the RF, RG, and RW systems, respectively (**Figure 7**). The RR system scored 1.25 in each of food security and economic profitability. These values were 0.13 and 0.33 higher than those of the RW system; 0.75 and 1.00 higher than those of the RG system; and 1.00 and 1.00 higher than those of the RF system. In resource utilization, the RG, RW and RR systems were 0.44, 1.00, and 0.81 higher than the RF systems. Regarding environmental impact, the RF, RW and RR systems received identical scores, which were 0.13 lower than those of the RG system. According to the survey data, the SPI values for the RF system were consistent with the experimentally recorded values, whereas those for the RG and RR systems were 0.19 and 0.13 lower than the recorded values, respectively. By contrast, the SPI value for the RW system was 0.29 higher. Notably, among the surveyed systems, the RR system achieved the highest scores across all three aspects except for the environmental impact, while the RW system only matched its score in the food security.

**Figure 7:**
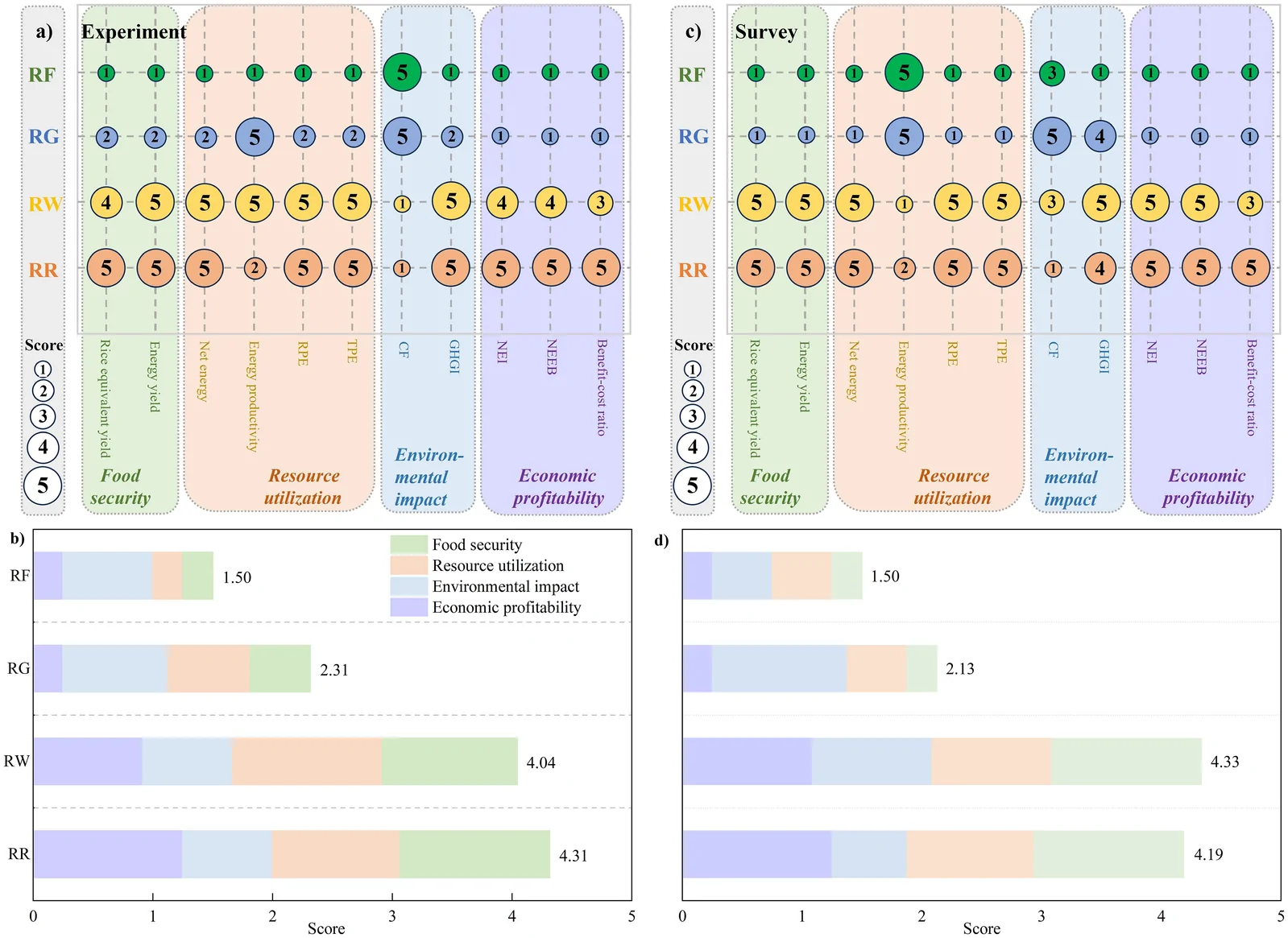
The sustainability performance index **(b, d)** and scores of each aspect **(a, c)** of the four rice-based cropping systems. RF, rice−fallow; RG, rice−green manure; RW, rice−wheat; RR, rice−rapeseed; CF, carbon footprint; GHGI, carbon emission intensity; RPE, radiation production efficiency; TPE, accumulated temperature production efficiency; NEEB, net ecosystem economic budget; NEI, net economic income.

## 4 Discussion

### 4.1 Food security and economic profitability

Increasing crop productivity per unit area has consistently been a key measure to safeguard national food security. In this study, both field experiments and farmer surveys indicated that the RR and RW systems had higher rice equivalent yield than the RF and RG systems. This finding is consistent with that of a number of studies, which have mainly attributed the increase to higher yields from winter crops (Huang et al., 2022; Li et al., 2020; Tao, 2020). Notably, the rice yields in the RF system were significantly lower than those of other systems in both experiments and surveys, mainly attributed to lower total dry matter weight and reduced nitrogen uptake. Both winter crop residue return and application of green manure can increase the yield of the subsequent rice crop. Returning straw to the fields significantly increases the organic carbon, total nitrogen, and nutrient content (NO_3_^-^, NH_4_^+^, and available phosphorus) in both the topsoil and subsoil, which increases nutrient supply and uptake, thereby stimulating plant development (Thangarajan et al., 2013; Yang et al., 2022; Ma et al., 2024). This study found that the N content in rice straw under the RG, RW, and RR systems at maturity was significantly higher than that under the RF system (**Supplementary Table S1**). Meanwhile, the incorporation of winter crop straw and the application of green manure enhanced soil C and N contents, thereby promoting nutrient supply and uptake by rice plants, which ultimately led to increased grain yield. The rice equivalent yield of the RR system was significantly higher than that of the RW system, primarily owing to the greater market price of rapeseed over wheat grain. However, the difference in energy yield between the two systems was not significant in either the experiments or the surveys. This is mainly because the energy equivalent coefficient of rapeseed is nearly twice that of wheat grain, which offsets the grain yield difference and results in a similar net energy yield. However, previous studies have concluded differently from ours, which concluded that the RW system has a higher energy yield than the RR system (Huang et al., 2022). This inconsistency could be explained by differing cultivation methods and climatic conditions.

Whether a cropping system can be widely accepted by farmers depends on its capacity to generate favorable economic benefits for them. Undoubtedly, the cultivation of winter crops increases annual agricultural inputs, thereby raising production costs. With respect to carbon cost, relative to the RF system, the carbon costs per unit yield increment for the RG, RW, and RR systems were 4.2%, 38.2%, and 36.9% higher, respectively. Interestingly, although the RW and RR systems were associated with higher agricultural and carbon costs than the RF and RG systems, they exhibited superior performance in key economic profitability indicators (NEI, NEEB, and benefit-cost ratio), a result driven by their higher rice equivalent yields (**Table 5**). These findings indicate that, compared to winter fallow and winter green manure systems, the cultivation of wheat or rapeseed in winter can not only increase annual grain yield and alleviate the pressure of food demand from population growth, but also enhance the annual economic benefits of the rice-based cropping system, provided that appropriate cultivation practices are implemented.

### 4.2 Resource utilization

In the context of current resource scarcity, improving resource use efficiency is crucial for promoting sustainable agricultural development (Burgess et al., 2022). In this study, the cultivation of winter crops increased agricultural inputs, especially in nitrogen fertilizer, diesel fuel, and electricity, resulting in greater energy inputs in the RW and RR systems than in the RF and RG systems. This is consistent with previous studies (Ghosh et al., 2022; Ji et al., 2024). Yadav et al. (2017) concluded that fertilizers (N, P, K and micronutrients) were the major contributors, accounting for 47% of the total energy inputs. However, the higher energy output resulted in significantly higher net energy in the RW and RR systems compared to the RF and RG systems, rather than being lower (**Table 4**). In addition, the RR and RW systems demonstrated significantly greater energy productivity than the RF and RG systems in the experiments. These results are supported by several studies, which reported that rice-based cropping systems with higher energy input may have higher energy output and utilization (Biswas et al., 2006; Chaudhary et al., 2009; Dey et al., 2024).

Crop yield is determined by the availability of light-thermal resources during the growth period and the crop’s use efficiency of these resources (Chen et al., 2016; Zhang et al., 2014). In countries with limited arable land per capita and shortages of grain and oil crops, such as China, winter fallow or the cultivation of non-grain-oil crops would undoubtedly lead to a waste of land and light-thermal resources. In this study, the RR and RW systems demonstrated a significant advantage in both RPE and TPE compared to the RF and RG systems, leading to a greater annual yield. This result indicates that the cultivation of wheat or rapeseed in winter can increase the multiple cropping index and enhance the use efficiency of light-thermal resources, thereby improving the annual yield per unit land area. This plays a significant role in mitigating the conflict between limited arable land per capita and the high demand for grain and oil crops.

### 4.3 Environmental impact

Our findings indicate that, in both experiments and surveys, the winter, rice season, and annual CF of the RW and RR systems were significantly higher than those of the RF and RG systems. Specifically, both RW and RR systems exhibited greater direct and indirect GHG emissions during the winter season compared with the RF and RG systems. The increase in direct GHG emissions was mainly due to elevated N_2_O emissions. Studies have shown that nitrogen application to winter crops increases the abundance of ammonium oxidizers and denitrifiers, thereby promoting N_2_O emissions (Duan et al., 2022). Additionally, the additional application of nitrogen fertilizer significantly increases the nitrogen substrate required for nitrification and denitrification, leading to elevated N_2_O emissions (Ma et al., 2010). Previous studies showed that incorporating winter crops increases soil pH, and higher soil pH correlates with elevated nitrification rates, ultimately leading to increased N_2_O emissions (Xu et al., 2021; Xue et al., 2020). The rise in indirect GHG emissions is undoubtedly attributable to greater input consumption. In contrast, differences in the CF among four systems during the rice season were primarily reflected in direct GHG emissions. Compared with the RG and RF systems, the higher direct GHG emissions of RW and RR systems in the rice season was largely ascribed to increased CH_4_ emissions. After being incorporated into the soil, wheat and rapeseed straws decompose through interactions with soil microorganisms and enzymes, providing abundant available substrates for methanogens (Stemmer et al., 2008; Wingeyer et al., 2012). This stimulates the growth of methanogens and further enhances CH_4_ emissions during the rice season (Wassmann et al., 2000). Simultaneously, straw decomposition consumes oxygen, accelerating the formation of anaerobic conditions favorable for methanogenic activity and further increasing CH_4_ emissions (Liu et al., 2014; Yao et al., 2013). Notably, the lower CF of the RR system than the RW system was primarily driven by the rice season, during which CH_4_ emissions were the most significant factor contributing to the difference, rather than N_2_O or indirect emissions. Previous studies also showed that the contribution to CF is much larger for CH_4_ emissions than for N_2_O (Raheem et al., 2019; Sun et al., 2021; Xu et al., 2024b). Generally, a high carbon/nitrogen (C/N) ratio in straw is associated with increased CH_4_ emission (Raheem et al., 2019). Crop growth influences carbon input and nitrogen uptake, thereby affecting CH_4_ emissions. The C/N ratio affects the organic carbon content while simultaneously enhancing the activity of methanogenic archaeal gene products, thus ensuring maximum CH_4_ emission. The low C/N ratio of green manure results in lower CH_4_ emissions during the rice season compared to those under the RW and RR systems (**Supplementary Table S1**). Our study revealed that rapeseed straw possesses a C/N ratio that is 49.5% higher than that of wheat straw, a finding consistent with the observations of Okubo et al. (2016). Because the higher lignin and cellulose content in rapeseed straw results are slow to decompose in the soil, which has limited effect on increasing the soil C/N ratio (Liang et al., 2023).

Our study found that planting wheat and rapeseed during the winter season increased annual GHG but reduced the annual GHGI in both experiments and surveys. The lower GHGI of the RR and RW systems compared to the RF and RG systems resulted mainly from higher yields, while the even lower GHGI of the RR system relative to the RW system was due to its higher yield and lower CF. Consequently, from the perspective of environmental sustainability, despite the higher GHG emissions resulting from the inclusion of winter crops in the RR and RW systems, this drawback was offset by the high yields obtained across both seasons. This results in a greater reduction in GHGI, establishing them rice-based cropping systems capable of balancing grain production and environmental impact. Future efforts may focus on optimizing crop management practices, such as optimizing nitrogen application rates, adopting controlled or slow-release fertilizers, and implementing alternate wetting and drying irrigation, to mitigate GHG emissions from the RR and RW systems.

### 4.4 Overall sustainability performance and limitations

The study summarized the essential demands for agriculture to four aspects in the sustainability performance, and calculated the SPI for a comprehensive comparison using a weighted average approach. The RR and RW systems achieved higher SPI values than the other systems, owing to their superior performance in all aspects except for environmental impact. Although the RG system scored 0.13 points higher in environmental impact than the RR and RW systems in the experiments, its lower scores in food security, resource utilization and economic profitability resulted in a lower SPI. In the surveys, the RW and RR systems, despite its lower environmental scores, secured a higher SPI because its strengths in other aspects offset this disadvantage. The worse performance in food security, resource utilization and economic profitability aspects of the RF system across both surveys and experiments resulted in the lowest SPI.

Throughout, there are some limitations and uncertainties in this study. Firstly, this study was only considered GHG emissions in environmental assessment, due to the difficulty in accurately obtaining indicators such as nitrogen leaching, water eutrophication, and soil degradation under field conditions, these factors were not included in the SPI of this study. Future research will further incorporate these indicators to enable a more accurate evaluation of SPI values across different systems. Secondly, since the SPI values in this study were calculated from a comprehensive perspective, the four components were assigned equal weights, which may overlook multi-stakeholder perspectives. For instance, farmers primarily focus on profitability, whereas environmentalists emphasize environmental impacts. Furthermore, the survey revealed pronounced challenges pertaining to elevated labor costs and labor shortages. In response to these issues, the adoption of modern agricultural practices, such as UAV (unmanned aerial vehicle)-based fertilization and pesticide application, alongside automated transplanting and harvesting machinery, represents a viable approach for field cultivation. Finally, this study was conducted over a limited duration of only two experimental cycles. Although this meets the time constraints for field research and is supplemented by surveys, it remains a limitation. Therefore, long-term positioning experiments are planned to further validate these findings.

## 5 Conclusions

This study conducted a two-year field experiment and a farmer survey to comprehensively assess the performance of four major rice-based cropping systems in the Yangtze River Basin of China. Compared to the RF and RG systems, the RR and RW systems exhibited higher rice equivalent yield, energy yield, net energy, RPE, TPE, NEI, NEEB, and lower GHGI, although they had greater CF. Overall, the RR and RW systems demonstrated superior SPI compared to the RF and RG systems. This enhanced performance was largely attributable to their higher scores in food security, resource utilization, and economic profitability. Our findings demonstrate that incorporating wheat or rapeseed in winter leads to significant gains in annual productivity, resource use efficiency, and economic benefits of rice-based cropping systems per unit land area, which contributes to sustainable agricultural development. The study results can provide a practical reference for selecting sustainable rice-based cropping systems in the Yangtze River Basin of China with comparable ecological and socio-economic conditions. Furthermore, it is recommended to enhance the widespread adoption of high-yield and high-quality varieties, new machinery suitable for large-scale production, controlled-release and efficiency-enhanced fertilizers, and corresponding cultivation techniques. These integrated approaches could substantially enhance the productivity, resource use efficiency, and economic benefits of rice-based cropping systems while mitigating environmental impacts, particularly during the rice-growing season.

## Data Availability

The original contributions presented in the study are included in the article/**Supplementary Material**. Further inquiries can be directed to the corresponding author.
