# Supplementary material for: Strategies for sustainable crop production in the Yangtze River Basin of China: insights from field experiments and farm surveys
Source: Front Plant Sci. 2026 Oct 2;17:1933498. doi: 10.3389/fpls.2026.1933498 (PMC13671957; doi:10.3389/fpls.2026.1933498)
Supplement: Supplementary file 1 [file SupplementaryFile1.docx]

**Supplementary material Legends**

Fig. S1. Daily solar radiation (MJ m^-2^ d^-1^), daily precipitation (mm), daily maximum temperature (T_max_, ℃), daily minimum temperature (T_min_, ℃) and daily average temperature (T_avg_, ℃) in the 2022–2024..

Fig. S2. Crop growth periods of four rice-based cropping systems during 2022–2023 and 2023–2024 cycles. RF, rice−fallow; RG, rice−green manure; RW, rice−wheat; RR, rice−rapeseed.

Fig. S3. Calibration (a) and validation (b) of the DNDC model. RF, rice−fallow; RG, rice−green manure; RW, rice−wheat; RR, rice−rapeseed.

Table S1. The environmental indicators of straw at maturity stage in four rice-based cropping system experiments.

Table S2. The complete questionnaire of four rice-based cropping system.

Table S3. CO_2_ equivalent coefficients of various agricultural inputs.

Table S4. Input information of model parameters.

Table S5. Input information after model calibration of four rice-based cropping systems.

Table S6. Normality assessment in survey of energy yield.

**Fig. S1.** **Daily solar radiation (MJ m^-2^ d^-1^), daily precipitation (mm), daily maximum temperature (T_max_, ℃), daily minimum temperature (T_min_, ℃) and daily average temperature (T_avg_, ℃) in the 2022**–**2024.**

**Fig. S2. Crop growth periods of four rice-based cropping systems during 2022**–**2023 and 2023**–**2024 cycles.** RF, rice−fallow; RG, rice−green manure; RW, rice−wheat; RR, rice−rapeseed.

**Fig. S3. Calibration (a) and validation (b) of the DNDC model.** RF, rice−fallow; RG, rice−green manure; RW, rice−wheat; RR, rice−rapeseed.

**Table S1. The environmental indicators of straw at maturity stage in four rice-based cropping system experiments.**

| Year | System | Rice straw | | |  |  | Winter straw | | | |  | |
| --- | --- | --- | --- | --- | --- | --- | --- | --- | --- | --- | --- | --- |
|  |  | N | C | C/N |  | N | | C | C/N | | |  |
| (Y) | (S) | kg ha^-1^ | kg ha^-1^ | % |  | kg ha^-1^ | | kg ha^-1^ | % | | |  |
| 2022–2023 | RF | 63.7 b | 2622.3 a | 41.2 a |  | / | | / | / | | |  |
|  | RG | 84.0 ab | 3320.3 a | 40.5 a |  | 54.2 a | | 885.6 b | 16.6 c | | |  |
|  | RW | 77.9 ab | 3068.9 a | 39.8 a |  | 37.2 b | | 1446.9 a | 39.0 b | | |  |
|  | RR | 103.8 a | 3428.0 a | 33.2 b |  | 28.6 b | | 1602.6 a | 56.3 a | | |  |
| 2023–2024 | RF | 127.7 b | 4037.3 a | 32.3 ab |  | / | | / | / | | |  |
|  | RG | 137.2 ab | 4438.3 a | 33.0 a |  | 35.9 a | | 882.0 b | 26.0 b | | |  |
|  | RW | 156.4 a | 4544.4 a | 29.1 ab |  | 18.4 b | | 816.6 b | 45.7 b | | |  |
|  | RR | 163.7 a | 4405.7 a | 26.9 b |  | 22.0 ab | | 1484.3 a | 70.3 a | | |  |
| Analysis of variance | | | | | | | | | |  |  |  |
| S |  | * | ns | ** |  | ** | | ** | ** | | |  |
| Y |  | ** | ** | ** |  | ** | | ** | * | | |  |
| S×Y |  | ns | ns | ns |  | ns | | ** | ns | | |  |

RF, rice−fallow; RG, rice−green manure; RW, rice−wheat; RR, rice−rapeseed; N, nitrogen accumulation; C, carbon accumulation. Within a column for each year, means followed by different letters are significantly different according to LSD (0.05). ns, not significant at 0.05 probability level; * and ** are significant at the 0.05 and 0.01 probability levels, respective.

**Table S2. The complete questionnaire of four rice-based cropping system.**

| Site | Number |  | Address | City District / Country | | |
| --- | --- | --- | --- | --- | --- | --- |
|  | Village Name of farmers |  | Contact Information |  | Coordinates of the Field |  |
| Rice  season | Variety name |  | Variety type |  | Cultivated area |  |
|  | Sowing date (year-month-day) |  | Transplanting date (year-month-day) |  | Harvest date (year-month-day) |  |
|  | Seeding rate (year-month-day) |  | Seedling raising method |  | Transplanting method |  |
|  | N rate (kg ha^-1^) |  | P_2_O_5_ rate (kg ha^-1^) |  | K_2_O rate (kg ha^-1^) |  |
|  | Total insecticide amount (kg ha^-1^) |  | Total fungicide amount (kg ha^-1^) |  | Total herbicide amount (kg ha^-1^) |  |
|  | Mechanical operation time (h ha^-1^) |  | Total labor input (h ha^-1^) |  | Electricity input (kWh ha^-1^) |  |
|  | Irrigation frequency (times ha^-1^) |  | Cost ($ ha^-1^) |  | Yield (kg ha^-1^) |  |
| Green manure season | Variety name |  | Variety type |  | Cultivated area |  |
|  | Sowing date (year-month-day) |  | Seeding rate (year-month-day) |  | Seeding method |  |
|  | Mechanical operation time (h ha^-1^) |  | Total labor input (h ha^-1^) |  | Cost ($ ha^-1^) |  |
| Wheat / Rapeseed season | Variety name |  | Variety type |  | Cultivated area |  |
|  | Sowing date (year-month-day) |  | Seeding rate (year-month-day) |  | Harvest date (year-month-day) |  |
|  | Seeding method |  | Electricity input (kWh ha^-1^) |  | Irrigation frequency (times ha^-1^) |  |
|  | N rate (kg ha^-1^) |  | P_2_O_5_ rate (kg ha^-1^) |  | K_2_O rate (kg ha^-1^) |  |
|  | Total insecticide amount (kg ha^-1^) |  | Total fungicide amount (kg ha^-1^) |  | Total herbicide amount (kg ha^-1^) |  |
|  | Electricity input (kWh ha^-1^) |  | Total labor input (h ha^-1^) |  | Cost ($ ha^-1^) |  |
|  | Yield (kg ha^-1^) | |  |  | |  |

**Table S3. CO_2_ equivalent coefficients of various agricultural inputs.**

| Items | Unit | Emission factor  (CO_2_-eq kg unit^-1^) | Reference |
| --- | --- | --- | --- |
| Machinery | h | 3.32 | Kumar et al., 2021 |
| Diesel | kg | 4.99 | CLCD, 2021 |
| Nitrogen (N) | kg | 1.53 | CLCD, 2021 |
| Phosphorus (P_2_O_5_) | kg | 1.63 | CLCD, 2021 |
| Potassium (K_2_O) | kg | 0.65 | CLCD, 2021 |
| Electricity | kWh | 1.23 | CLCD, 2021 |
| Insecticide | kg | 16.61 | Ecoinvent, 2010 |
| Fungicide | kg | 10.57 | Ecoinvent, 2010 |
| Herbicide | kg | 10.15 | Ecoinvent, 2010 |
| Rice seed | kg | 1.84 | Ecoinvent, 2010 |
| Wheat seed | kg | 1.16 | Liu et al., 2014 |
| Rapeseed | kg | 0.83 | Gan et al., 2012 |
| Labor | h | 0.036 | Liu et al., 2014 |

**Table S4. Input information of model parameters.**

| Data | Crop physiological and phenological parameters | Parameter value |
| --- | --- | --- |
| Climate | Site | Chao hu |
|  | Latitue | 31.66 |
|  | Simulated years | 3 |
|  | Climate files fornat | J day, MaxT, MinT, Prec, Radi |
|  | N concentration in rainfall (mg N L^-1^ or ppm) | 0 |
|  | Atmospheric background NH_3_ concentration  (ug N m^-3^) | 0.06 |
|  | Atmospheric background CO_2_ concentration (ppm) | 350 |
|  | Annual increase rate of atmospheric CO_2_ concentration (ppm yr^-1^) | 0 |
| Soil | Land use | Rice paddy field |
|  | Texture | Silty clay loam |
|  | Clay fraction | 0.34 |
|  | Bulk density | 0.8249 |
|  | Field capacity (wfps) | 0.55 |
|  | Conductitivity (m hr^-1^) | 0.015 |
|  | Soil pH | 6.44 |
|  | Wilting point (wfps) | 0.26 |
|  | Porosity | 0.477 |
|  | Depth of water-retention layer (m) | 9.99 |
|  | Drainage efficiency | 1 |
|  | SOC at surface soil (0 cm ~10 cm, kg C kg^-1^) | 0.05225 |
|  | Initial nitrate concentration (mg N kg^-1^) | 2.62 |
|  | Initial ammonium concentration (mg N kg^-1^) | 7.39 |
|  | Microbial activity index | 1 |
| Cropping | Slope | 0 |
|  | Soil salinity index | 0 |
|  | Rain water collection index | 1 |
|  | Total simulated years | 3 |
|  | Number of cropping systems | 4 |
|  | Cropping system # | 1# 2# 3# 4# |
|  | Years this cropping system last for | 3 |
|  | Years of a cycle within this cropping system | 3 |

**Table S5. Input information after model calibration of four rice-based cropping systems.**

| Management | Parameters | RF | RG | | RW | | RR | |
| --- | --- | --- | --- | --- | --- | --- | --- | --- |
| Crop | Crops | 2 | 2 | | 2 | | 2 | |
|  | Crop type | Paddy rice | Paddy rice | Green manure | Paddy rice | Spring wheat | Paddy rice | Rapeseeds |
|  | Cover | Not | Not | Not | Not | Not | Not | Not |
|  | Planting date | 6/20 | 6/20-10/14 | 10/14 | 6/20 | 11/10 | 6/20 | 10/14 |
|  | Harvest date | 9/30 | 9/30 | 0 | 9/30 | 5/30 | 9/30 | 5/21 |
|  | Transplanting | Yes | Yes | No | Yes | No | Yes | No |
|  | Plant | No | No | No | No | Yes | No | Yes |
|  | Harvest mode | This year | This year | 0 | This year | Next year | This year | Next year |
|  | Max biomass production  (kg C ha^-1^ yr^-1^) | 9545/5187/6225/30/917 | 10807/5026/7640/26791 | 0/0/0/0 | 10024/3580/5226/16969 | 7843/3087/4693/2470 | 10356/4045/6027/20024 | 4216/5492/5960/48459 |
|  | Biomass fraction  (grain / leaf / stem / root) | 0.184/0.1/0.12/0.596 | 0.215/0.1/0.152/0.533 | 0.1/0.25/0.25/0.4 | 0.28/0.1/0.146/0.474 | 0.508/0.22/0.504/0.16 | 0.256/0.1/0.149/0.495 | 0.216/0.34/0.369/0.03 |
|  | C/N (grain / leaf / stem / root) | 22/41/40/41 | 26/40/40/41 | 1/1/1/1 | 25/40/39/41 | 17/39/38/40 | 24/33/33/41 | 19/56/56/71 |
|  | Thermal degree days | 2000 | 2000 | 0 | 2000 | 1300 | 2000 | 1800 |
|  | Water (g water g^-1^ DM) | 508 | 508 | 0 | 508 | 300 | 508 | 300 |
|  | N fixation index | 1.05 | 1.05 | 1 | 1.05 | 1 | 1.05 | 1 |
|  | Optimum temperature (℃) | 25 | 25 | 20 | 25 | 22 | 25 | 25 |
|  | Vascularity | 1 | 1 | 0 | 1 | 1 | 1 | 1 |
| Tillage | Applications | 3 | 4 | / | 3 | 2 | 3 | 2 |
|  | Month / day | 5/30 6/20 9/30 | 5/30 6/20 9/30 | 10/14 | 5/30 6/20 9/30 | 11/10 5/30 | 5/30 6/20 9/30 | 10/14 5/21 |
|  | Tilling method | Ploughing with moldboard, 20 cm crop terminating till | | | | | | |

| Fertilization | Applications in this year # | 3 | 3 | 0 | 3 | 2 | 3 | 2 |
| --- | --- | --- | --- | --- | --- | --- | --- | --- |
|  | Application date | 6/21 7/10 8/16 | 6/21 7/10 8/16 | 0 | 6/21 7/10 8/16 | 11/14 1/25 | 6/21 7/10 8/16 | 10/17 1/25 |
|  | Application depth (cm) | 3 | 3 | 0 | 3 | 5 | 3 | 5 |
|  | Urea (kg ha^-1^) | 380 76 76 | 380 76 76 | 0 | 380 76 76 | 380 76 | 380 76 76 | 456 130 |
|  | Phosphate (kg ha^-1^) | 175 0 0 | 175 0 0 | 0 | 175 0 0 | 175 0 | 175 0 0 | 230 0 |
| Manure Amendment | Applications in this year # | 1 | 1 | 1 | 1 | 1 | 1 | 1 |
|  | Month / day | 9/30 | 9/30 | 10/14 | 9/30 | 9/30 | 9/30 | 9/30 |
|  | Manure type | Straw | Straw | Green manure | Straw | Straw | Straw | Straw |
|  | Solid C/N ratio | 70 | 70 | 25 | 70 | 70 | 70 | 70 |
| Irrigation | Based on ligation events | / | / | / | / | 2 | / | 2 |
|  | Month / Day | / | / | / | / | 1/24 3/29 | / | 3/11 4/23 |
|  | Amount (cm) | / | / | / | / | 20 | / | 20 |
|  | Method | / | / | / | / | Furrow | / | Furrow |
| Fooding | Applications | 2 | 2 | / | 2 | / | 3 | / |
|  | Start date | 6/20 7/20 | 6/20 7/20 | / | 6/20 7/20 | / | 6/20 7/20 | / |
|  | 结束日期 End date | 7/21 9/20 | 7/21 9/20 | / | 7/21 9/20 | / | 7/21 9/20 | / |
|  | Continuous or Alternative wet and dry | All | All | / | All | / | All | / |
|  | Leaking rate (mm day^-1^) | 1.5 | 1.5 | / | 1.5 | / | 1.5 | / |
| Grazing or cutting | Number of cuts | 1 | 1 | / | 1 | 1 | 1 | 1 |
|  | Month / day | 9/30 | 9/30 | / | 9/30 | 5/30 | 9/30 | 5/21 |
|  | Cut part | Grain Leaf Stem | | | | | | |
|  | Cut fraction (0-1) | 0.8 | 0.8 | / | 0.8 | 0.8 | 0.8 | 0.8 |

RF, rice−fallow; RG, rice−green manure; RW, rice−wheat; RR, rice−rapeseed.

**Table S6. Normality assessment in survey of energy yield.**

| Test | Kolmogorov-Smirnov | | | |  | Shapiro-Wilk | | | |
| --- | --- | --- | --- | --- | --- | --- | --- | --- | --- |
|  | Statistic | df | p-value | Conclusion |  | Statistic | df | p-value | Conclusion |
| RF | 0.152 | 50 | 0.105 | Normal |  | 0.966 | 50 | 0.164 | Normal |
| RG | 0.202 | 30 | 0.103 | Normal |  | 0.946 | 30 | 0.131 | Normal |
| RW | 0.097 | 55 | 0.200 | Normal |  | 0.971 | 55 | 0.217 | Normal |
| RR | 0.095 | 55 | 0.200 | Normal |  | 0.971 | 55 | 0.217 | Normal |

A p-value of less than 0.05 indicates a significant deviation from normality, whereas a p-value greater than 0.05 suggests that the data follow a normal distribution. RF, rice−fallow; RG, rice−green manure; RW, rice−wheat; RR, rice−rapeseed.

**References**

Kumar, A., Rana, K.S., Choudhary, A.K., Bana, R.S., Sharma, V.K., Prasad, S., Gupta, G., Choudhary, M., Peadhan, A., Rajpoot, S.K., Kumar, A., Kumar, A., Tyagi, V. (2021). Energy budgeting and carbon footprints of zero-tilled pigeonpea-wheat cropping system under sole or dual crop basis residue mulching and Zn-fertilization in a semi-arid agro-ecology. *Energy*, 231, 120862. doi:10.1016/j.energy.2021.120862.

Chinese Core Life Cycle Database (CLCD), China. (2021). Core Life Cycle Database v0.7. http://www.ike-global.com/#/products-2/chinese-lca-database-clcd.

Ecoinvent, Ecoinvent Life Cycle Inventory Database v2.2. Swiss Centre for Life Cycle Inventories, 2010. http://www.ecoinvent.ch/. (Accessed 7 November 2010).

Liu, X.H., Xu, W.X., Li, Z.J., , Chu, Q.Q. Yang, X.L., Chen, F. (2014). The missteps, improvement and application of carbon footprint methodology in farmland ecosystems with the case study of analyzing the carbon efficiency of China’s intensive farming. *Chinese Journal of Agricultural Resources and Regional Planning*, 34: 1–11. doi:10.7621/cjarrp.1005-9121.20130601 (in Chinese with English abstract).

Gan, Y., Liang, C., Campbell, C.A., Zentner, R.P., Lemke, R.L., Wang, H., Yang, C. (2012). Carbon footprint of spring wheat in response to fallow frequency and soil carbon changes over 25 years on the semiarid Canadian prairie. European Journal of Agronomy, 43: 175–184. doi:10.1016/j.eja.2012.07.004.
